# Supplementary material for: Stacking Model‐Based Classifiers for Dealing With Multiple Sets of Noisy Labels
Source: Biom J. 2025 Mar 12;67(2):e70042. doi: 10.1002/bimj.70042 (PMC11898607; doi:10.1002/bimj.70042)
Supplement: Supplementary file 1 — Supporting Information [file BIMJ-67-e70042-s001.zip › density-based-ensemble-model-main/competitors_real_data/netlab-master/netlab-master/help/mdnfwd.htm]

Netlab Reference Manual mdnfwd


# mdnfwd

## Purpose

Forward propagation through Mixture Density Network.

## Synopsis

```
mixparams = mdnfwd(net, x)
[mixparams, y, z] = mdnfwd(net, x)
[mixparams, y, z, a] = mdnfwd(net, x)
```

## Description

`mixparams = mdnfwd(net, x)` takes a mixture density network data
structure `net` and a matrix `x` of input vectors, and forward
propagates the inputs through the network to generate a structure
`mixparams` which contains the parameters of several mixture models.
Each row of `x` represents
one input vector and the corresponding row of the matrices in `mixparams`
represents the parameters of a mixture model for the conditional probability
of target vectors given the input vector. This is not represented as an array
of `gmm` structures to improve the efficiency of MDN training.

The fields in `mixparams` are

```
  type = 'mdnmixes'
  ncentres = number of mixture components
  dimtarget = dimension of target space
  mixcoeffs = mixing coefficients
  centres = means of Gaussians: stored as one row per pattern
  covars = covariances of Gaussians
  nparams = number of parameters
```

`[mixparams, y, z] = mdnfwd(net, x)` also generates a matrix `y` of
the outputs of the MLP and a matrix `z` of the hidden
unit activations where each row corresponds to one pattern.

`[mixparams, y, z, a] = mlpfwd(net, x)` also returns a matrix `a`
giving the summed inputs to each output unit, where each row
corresponds to one pattern.

## See Also

`mdn`, `mdn2gmm`, `mdnerr`, `mdngrad`, `mlpfwd`

---

**Pages:**
Index


---

Copyright (c) Ian T Nabney (1996-9)

David J Evans (1998)
